# Effectiveness of a hysteroscopic tissue removal system device for hysteroscopic myomectomy on patients’ quality of life: a randomized clinical trial

**DOI:** 10.1186/s12905-023-02707-3

**Published:** 2023-10-17

**Authors:** Teresa Tam, Lourdes Juarez

**Affiliations:** 1 Department of Obstetrics and Gynecology, Ascension Saint Francis Hospital, 355 Ridge Ave, Evanston, IL 60202 USA; 2 Department of Obstetrics and Gynecology Edward-Elmhurst Medical Group, 100 Spalding Dr #406, Naperville, IL 60540 USA

**Keywords:** Hysteroscopy, Leiomyoma, Abnormal uterine bleeding, Quality of life, Hysteroscopic myomectomy, Heavy menstrual bleeding, Uterine fibroid symptom and health-related quality of life (UFS-QOL) questionnaire

## Abstract

**Purpose:**

To evaluate the quality of life in patients treated for submucosal leiomyomas after hysteroscopic myomectomy compared to medical therapy. This is the first prospective randomized analysis comparing outcomes of medical therapy versus hysteroscopic myomectomy using the TruClear™ hysteroscopic tissue removal system to treat heavy menstrual bleeding from submucosal leiomyoma(s).

**Methods:**

Setting: private practice and community-based hospital; subjects: female patients with symptomatic submucosal leiomyomas from 2014 to 2017. A total of 69 patients enrolled, with 47 completed. Statistical analysis used: randomization, linear mixed-effects modeling, hypothesis testing, and intent-to-treat analysis. Each patient was randomized to oral contraceptive pills/progesterone releasing intrauterine device or hysteroscopic myomectomy. Each patient was to complete the Uterine Fibroid Symptom and Health-related Quality of Life (UFS-QOL) questionnaire at baseline, one month, three months, and greater than or equal to six months after treatment. Main outcome measured: Primary outcome was the health-related quality of life (HR-QOL), as reflected from UFS-QOL scores. Contrasts were constructed from a linear mixed-effects model to compare the two treatment groups for changes from baseline in UFS-QOL scores.

**Results:**

UFS-QOL scores were similar at baseline between the two treatment groups. There was an overall improvement in all UFS-QOL scores within each group. Higher improvement scores were noted in the surgical group compared to the medical group for almost all UFS-QOL scores. At ≥ 6 months, in comparison to the medically managed patients, the most considerable score improvements for the surgical group were reported in HR-QOL concern, activities, self-consciousness and symptom severity scores having mean change scores (95% CIs) of 35.3, 28.9, 28.6, and 32.2, respectively.

**Conclusion:**

Although there was no statistical difference in the change degree of improvement of overall quality of life among patients with symptomatic submucosal leiomyomas who received medical or surgical treatments in the study, there were greater differences in improvements in health-related quality of life scores over time after surgical treatment.

## Introduction

Leiomyomas are the most common benign gynecological tumors diagnosed in women. They occur in approximately 25% of females and frequently occur in women with ages ranging from 30 to 40 [1]. Uterine leiomyomas occur more commonly in women of African descent, with black women being more than twice as likely to have a principal diagnosis of leiomyomas than white women at the time of hysterectomy [2, 3]. Marshall and colleagues identified a higher incidence of uterine leiomyomas in premenopausal women with larger and more numerous myomas among black women [4, 5]. Leiomyomas are more commonly seen in patients during their reproductive period and decrease in size with menopause. Although several risk factors such as age and premenopausal status increase the incidence of leiomyomas, the black race has been identified as the only factor consistently reported to increase the risk for leiomyomas [6].

Many symptoms are associated with leiomyomas, namely: abnormal uterine bleeding (AUB), heavy menstrual bleeding, dysmenorrhea, pelvic pain and pressure, urinary complaints of urgency and frequency, abdominal distention, and infertility [7]. Although these symptoms are often subjective, submucosal leiomyomas frequently cause AUB with heavy menstrual bleeding [8]. Many available treatment modalities are available. The choice of any treatment modality is dependent on a woman’s specific chief complaint, disease burden, and therapy decision.

Despite the multitude of minimally invasive surgical treatment options for uterine leiomyomas, hysteroscopic myomectomy remains a feasible option for managing submucosal leiomyomas [9–11]. Alternatively, abnormal uterine bleeding caused by leiomyomas can also be managed by medical therapy. Combined hormonal contraceptive, levonorgestrel-releasing IUD, tranexamic acid, oral or injectable progestin, and gonadotropin-releasing hormone (GnRH) are commonly used medications for reducing heavy menstrual bleeding associated with leiomyomas [12–15]. It is unclear whether medical therapy for treating AUB associated with leiomyomas provides a better quality of life compared to surgical management options.

The uterine fibroid symptom and quality-of-life questionnaire (UFS-QOL) is a validated tool to measure differences in fibroid-related symptoms and their impact on health-related quality of life (HR-QOL) while incorporating a patient perspective [16, 17]. The UFS-QOL provides a reproducible measurement of symptom severity and the impact of leiomyoma and post-treatment differences. Although several studies have looked at the impact of interventions on UFS-QOL, these analyses were primarily patient-reported outcomes after uterine-sparing procedures.

The aim of this study is to evaluate patients’ quality of life relating to treatment of submucosal leiomyomas by hysteroscopic myomectomy using a hysteroscopic tissue removal system compared to women managed medically. The medical therapy options in this study include oral contraceptive pills or levonorgestrel-containing intrauterine device (IUD).

## Methods

This is a randomized clinical trial of patients with symptomatic submucosal leiomyomas from 2014 to 2017. Oral and written informed consents were obtained from all patients in the study. Amita Saint Francis hospital in Evanston granted full Institutional in 2014 and Saint Joseph Hospital in Chicago granted full Institutional Review Board (IRB) approval in 2015. Periodic review of protocol that research does not involve greater than minimal patient risk was addressed as part of IRB submission approval. All methods were carried out in accordance with relevant guidelines and regulations. The study population includes women aged 18 and older with abnormal uterine bleeding and submucosal fibroids. Patients with Type 0, 1, or 2 submucosal myomas, assessed with a pelvic 2-D ultrasound, were recruited from the private practice of the attending surgeon, and included in the trial. Patients assigned to the medical therapy group includes use of combination oral contraceptive pills or a 52 mg levonorgestrel-releasing intrauterine device. Generic combined oral hormonal contraception of 20 mcg of ethinyl estradiol and norethindrone acetate was commonly prescribed. The surgical treatment group includes hysteroscopic myomectomy using the TruClear™ (Medtronic, Minneapolis, MN) hysteroscopic tissue removal system and procedure was performed in a community hospital setting. There was no endocrine priming done before treatment. Treatment was provided based on shared decision-making and procedure was aligned according to patient’s menstrual period and typically during the follicular phase of the menstrual cycle. The TruClear™ mechanical hysteroscopic tissue removal device used for measures 7.25 mm outer diameter.

Exclusion criteria included pregnancy, suspicion of uterine malignancy, and absence of submucosal leiomyoma(s) seen during planned hysteroscopic myomectomy. The study also excluded patients with active vaginal infection, women with contraindication to hysteroscopic myomectomy, and cognitively impaired patients who could not provide consent and adequately complete the questionnaire.

Patient demographics assessed include age, ethnicity, body mass index (BMI), parity, substance abuse, medical history, surgical history, and preoperative indication(s) for hysteroscopic myomectomy. A random allocation sequence was generated with randomization of the patients to two different assignment groups either medical or surgical treatment, using 1:1 allocation. The randomization scheme was created using a pseudo-random number generator (i.e., the PLAN procedure within the SAS software, version 9.4 (SAS Institute Inc., Cary, NC) and consisted of random permuted blocks, having variable blocks of either size 2 or 4, with a 1:1 allocation to the treatments. Once the patient signed the consent, the patient was randomized into one of the two arms through the drawing of a sealed, opaque envelope.

Patients completed the Uterine Fibroid Symptom and Health-related Quality of Life Questionnaire (UFS-QOL) at enrollment and at 1, 3, and greater than or equal to 6 months after treatment. Patients were asked to complete the UFS-QOL questionnaire form either during a follow-up visit in written format, follow up telephone encounter with recorded answers, mailed survey or online survey. The UFS-QOL questionnaire form has eight subscales measuring Health Related Quality of Life (HR-QOL). Higher scores (increased from baseline) for the subscales including concern, activities, energy/mood, control, self-consciousness, sexual function, and total score indicate improvement in HR-QOL. Lower scores (or decrease from baseline) in HR-QOL symptom severity subscale reflect improvement.

This trial based the sample size estimation on myomectomy patients’ UFS HR-QOL total score data by Spies et al. [17]. A UFS-QOL improvement from baseline to 6 months or more post-treatment of leiomyomas in the treatment arm using medication was anticipated to be similar to that reported by Spies et al. An improvement in UFS-QOL total score of approximately 40 points with a standard deviation of approximately 23 points was projected. We assumed a UFS-QOL improvement from baseline to 6 months or more post-treatment of leiomyomas in the treatment arm using surgical intervention with TruClear™ hysteroscopic tissue removal system to be 60 points. Thus, for sample size estimation of the trial, the expected difference between the two treatment arms was 20 points (i.e., 60 − 40) with a standard deviation of 23 points.

Based on these assumptions, a sample size of 24 patients per group provided 80% statistical power to detect a difference in the change from baseline to 6 months post-medical treatment in the UFS-QOL total scores of 20 points between the two treatment arms using a two-sided test with a significance level of 0.05. Therefore, it was planned to recruit at least 56 subjects for this study with an anticipated 20% dropout rate.

The primary analysis invoked an intent-to-treat paradigm, wherein all randomized subjects were included according to their randomized treatment assignment regardless of actual treatment received. A linear mixed-effects model was used to assess the differences between and within the two treatment arms (i.e., with and without TruClear™ hysteroscopic tissue removal system device) for the primary outcome of UFS-QOL total score. The fixed independent factors for the linear mixed-effects model included the treatment arm (with TruClear™ hysteroscopic tissue removal system device or medical therapy using hormonal contraceptive or levonorgestrel releasing IUD), time (baseline, 1, 3, and greater than or equal to 6 months), and the interaction of treatment and time. The mixed model included a random subject effect and a first order antedependence covariance structure for correlation among time points. From the linear mixed-effects model, comparisons were constructed to test the hypotheses of interest (e.g., change from baseline). The model does not drop subjects with incomplete (i.e., missing at random) data from the analysis. Differences in means and associated 95% confidence intervals (CIs) were used to quantify the effects’ magnitude. Similarly, separate linear mixed-effects models were used for each of the seven, secondary outcome UFS-QOL scores (i.e., concern, activities, energy/mood, control, self-conscious, sexual function, and symptom severity).

Although the sample size estimates considered subject dropout, efforts were made during the study to minimize any patient dropout. Study attrition did not appear to be an issue. All hypothesis tests were two-sided, and all analyses performed using SAS software, version 9.4. This trial is registered at ClinicalTrials.gov with clinical trial registry number NCT02934789, trial registration 17/10/2016, and URL:

https://register.clinicaltrials.gov/prs/app/template/EditRecord.vm?epmode=Edit&uid=U0002EJO&ts=6&sid=S0006MUC&cx=-uiayqz. The data repository set is available online at https://data.mendeley.com/datasets/xykswpsgmy/1.

## Results

Of the 69 patients enrolled in the study, 47 women completed the questionnaires either in person, by telephone, by mail or online. Twenty-four patients were from the medical intervention arm, and 23 patients had surgical intervention using the TruClear™ hysteroscopic tissue removal system. Of the 69 patients who completed their questionnaire, 42 completed by month 1, 36 completed by month 3, and 49 completed by month 6. However, two patients who completed the questionnaire at 6 months or greater interval did not meet the requirements of the study at the submission of the questionnaire. One patient had her IUD removed and another patient had an endometrial ablation performed prior to her questionnaire submission. Therefore, these two patients’ questionnaires were deemed invalid after their submission.

Patient demographics collected include age, body mass index (BMI), race, parity, history of medical problems and surgery(s) are shown in Table 1. The mean age is 39.9 years and BMI is 31.1 in the medical arm in comparison to mean age of 44.1 and BMI of 28.8 in the hysteroscopy assigned group. The race of the participants included black, white, Hispanic and others. The majority race of participants in the medical and hysteroscopy arm consisted of both black (39.1% medical, 29.2% hysteroscopy) and white (30.4% medical, 37.5% hysteroscopy) participants. Most patients in both groups were parous and have history of medical problems. Patients in the medical arm were more likely to have previous surgery (60.9%) versus patients assigned to the hysteroscopy arm (50%). Types of previous surgeries include cesarean section(s), gynecological (i.e., loop electrosurgical excision procedure, laparoscopy, myomectomy, hysteroscopy) and non-gynecological surgeries such as tonsillectomy, appendectomy, cholecystectomy, breast reduction, and orthopedic surgeries. None of the patients reported illicit drug usage or substance abuse. The most common preoperative indication for hysteroscopic myomectomy is abnormal uterine bleeding.

**Table 1:** Demographics of Study Participants

	Treatment Assignment	
**Characteristic**	Medical n (%)	Hysteroscopy n (%)
Age (years), mean ± SD	39.9 ± 8.5	44.1 ± 5.7
< 30 years	2 (8.7)	0 (0.0)
30–39 years	8 (34.8)	4 (16.7)
40–49 years	11 (47.8)	15 (62.5)
≥ 50 years	2 (8.7)	5 (20.8)
BMI (kg/m^2^), mean ± SD	31.1 ± 7.1	28.8 ± 6.1
< 25 kg/m^2^	5 (21.7)	7 (29.2)
25–29.9 kg/m^2^	9 (39.1)	8 (33.3)
≥ 30 kg/m^2^	9 (39.1)	9 (37.5)
Race		
Black	9 (39.1)	7 (29.2)
White	7 (30.4)	9 (37.5)
Hispanic	5 (21.7)	6 (25.0)
Asian	2 (8.7)	1 (4.2)
Other	0 (0.0)	1 (4.2)
Total Pregnancies		
None	5 (21.7)	8 (33.3)
1–2	10 (43.5)	12 (50.0)
≥ 3	8 (34.8)	4 (16.7)
History of Medical Problems		
None	10 (43.5)	6 (25.0)
Present	13 (56.5)	18 (75.0)
History of Surgery(s)		
None	9 (39.1)	12 (50.0)
Present	14 (60.9)	12 (50.0)
Medical Treatment		
Oral contraceptive pills	12 (50.0)	0 (0.0)
Levonorgestrel IUD	12 (50.0)	0 (0.0)

**Fig. 1:**
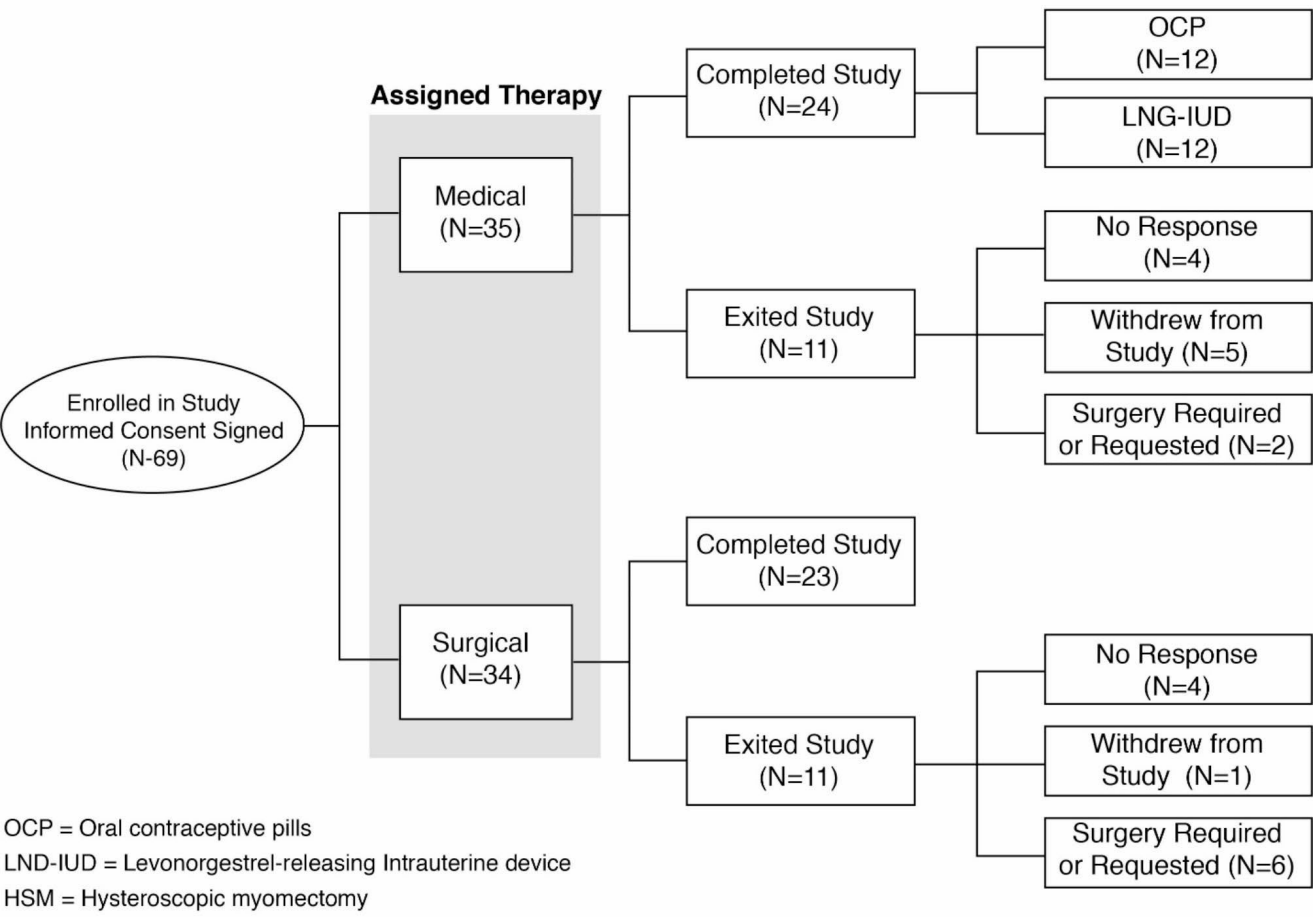
Subject Disposition Flowchart

A total of 22 patients dropped out of the study. The same number of patients (N = 11) dropped out from the surgical arm, like the number of patients who dropped out of the medication treatment arm (N = 11). The most common reasons for dropping out in either arm included lack of patient response, patient changing her mind after study enrollment, and request or requirement of (additional) surgery (Fig. 1). There were no adverse events reported from either group.

All patients in the surgical arm were treated under general anesthesia for hysteroscopic myomectomy in the outpatient setting. Although the surgical times were not measured, a vast majority of the surgeries were completed within an hour. Meanwhile, the patients in the medical arm did not undergo hysteroscopic assessment but only pelvic ultrasound measurement of their submucosal leiomyomas.

Between the two treatment groups, UFS-QOL scores were similar at baseline (Table 2). There was no evidence of any differences concerning changes in any UFS-QOL scores over time (Table 3). There was an overall improvement in all UFS-QOL scores within each group except for HR-QOL sexual function at one month for the surgical group. A supplemental table displays the fixed effects parameter estimates (i.e., coefficients) and standard errors (SEs), and the covariance parameter estimates and SEs for each of the 8 mixed models.

**Table 2:** UFS-QOL Scores at Baseline (Month 0)

UFS-QOL Score	Medication Mean ± SD	Surgery Mean ± SD
HRQL Concern Score	37.1 ± 33.4	38.6 ± 29.7
HRQL Activities Score	51.6 ± 33.1	51.7 ± 26.5
HRQL Energy/Mood Score	53.4 ± 31.6	56.3 ± 23.7
HRQL Control Score	49.2 ± 33.9	55.7 ± 25.5
HRQL Self-conscious Score	49.0 ± 34.4	49.2 ± 34.5
HRQL Sexual Function Score	42.5 ± 31.8	56.4 ± 36.3
HRQL Total Score	45.9 ± 29.3	51.3 ± 24.0
Symptom Severity Score	58.7 ± 23.4	58.2 ± 22.1

**Table 3:** Comparison of Changes in UFS-QOL Scores from Baseline

UFS-QOL Score	Month	Medication	Surgery	Medication vs. Surgery	
		Change from Baseline Mean (95% CI)	Change from Baseline Mean (95% CI)	Difference in Mean Changes (95% CI)	P-value
HRQL Concern Score	1	13.0 (0.3, 25.6)	14.1 (-0.3, 28.4)	-1.1 (-20.2, 18.0)	0.91
	3	15.5 (-0.5, 31.4)	31.5 (15.6, 47.5)	-16.1 (-38.6, 6.5)	0.16
	6	24.7 (11.9, 37.4)	35.3 (22.3, 48.2)	-10.6 (-28.8, 7.6)	0.25
HRQL Activities Score	1	6.6 (-3.3, 16.5)	8.9 (-2.3, 20.0)	-2.3 (-17.2, 12.6)	0.76
	3	11.7 (-1.1, 24.5)	22.5 (9.7, 35.4)	-10.8 (-29.0, 7.3)	0.24
	6	19.2 (6.7, 31.7)	28.9 (16.3, 41.5)	-9.7 (-27.4, 8.0)	0.28
HRQL Energy/Mood Score	1	6.9 (-1.4, 15.3)	8.7 (-0.6, 17.9)	-1.7 (-14.2, 10.8)	0.79
	3	12.6 (0.9, 24.3)	20.2 (8.3, 32.0)	-7.6 (-24.2, 9.1)	0.37
	6	17.5 (7.0, 28.1)	25.5 (14.8, 36.2)	-8.0 (-23.0, 7.0)	0.29
HRQL Control Score	1	12.7 (2.7, 22.7)	8.9 (-2.1, 19.8)	3.8 (-11.0, 18.7)	0.60
	3	13.6 (0.9, 26.4)	26.2 (13.3, 39.2)	-12.6 (-30.8, 5.6)	0.17
	6	20.4 (8.2, 32.5)	27.5 (15.1, 39.8)	-7.1 (-24.4, 10.3)	0.42
HRQL Self-conscious Score	1	13.7 (2.3, 25.2)	10.0 (-2.9, 23.0)	3.7 (-13.6, 21.0)	0.67
	3	10.0 (-4.3, 24.3)	19.3 (5.1, 33.6)	-9.4 (-29.6, 10.9)	0.36
	6	12.3 (-1.1, 25.7)	28.6 (15.0, 42.1)	-16.3 (-35.4, 2.8)	0.09
HRQL Sexual Function Score	1	7.2 (-3.5, 18.0)	-1.6 (-13.1, 9.9)	8.8 (-6.9, 24.6)	0.26
	3	19.8 (4.7, 35.0)	18.7 (3.8, 33.6)	1.1 (-20.1, 22.4)	0.92
	6	16.2 (1.0, 31.4)	19.5 (4.3, 34.7)	-3.3 (-24.8, 18.2)	0.76
HRQL Total Score	1	11.0 (1.5, 20.6)	8.9 (-1.2, 19.1)	2.1 (-11.9, 16.0)	0.77
	3	17.1 (6.1, 28.1)	25.2 (14.5, 35.9)	-8.1 (-23.5, 7.3)	0.30
	6	21.5 (10.7, 32.3)	27.1 (16.4, 37.7)	-5.6 (-20.7, 9.6)	0.47
Symptom Severity Score	1	-11.3 (-20.5, -2.1)	-14.6 (-25.2, -3.9)	3.3 (-10.8, 17.4)	0.64
	3	-19.2 (-30.3, -8.2)	-27.4 (-38.5, -16.3)	8.2 (-7.5, 23.8)	0.30
	6	-21.2 (-31.8, -10.6)	-32.2 (-43.0, -21.5)	11.0 (-4.1, 26.1)	0.15

Higher improvement scores were noted in the surgical group compared to the medical group for all UFS-QOL scores except for HR-QOL concern score at 1 month, HR-QOL self-conscious score at 1 month, HR-QOL sexual function score at 1 month and 3 months, and HR-QOL total score at 1 month. At ≥ 6 months, the most considerable score improvements for the surgical group were reported in HR-QOL concern, activities, self-conscious and symptom severity scores having mean change scores (95% CIs) of 35.3 (22.3–48.2), 28.9 (16.3–41.5), 28.6 (15.0–42.1), and 32.2 (21.5–43.0), respectively.

## Discussion

Hysteroscopic myomectomy remains one of the minimally invasive surgical options for treating AUB, typically excessive menstrual bleeding, in premenopausal women with submucosal leiomyomas. Although several treatment options are available, including medical interventions, these are primarily to treat heavy menstrual bleeding characteristic of women with submucosal leiomyomas. This study is the first prospective randomized analysis comparing outcomes of medical therapy versus hysteroscopic myomectomy using a hysteroscopic tissue removal system to treat heavy menstrual bleeding from submucosal leiomyoma(s).

Studies have shown that quality of life after hysteroscopic myomectomy was significantly better than after laparoscopic myomectomy due to shorter operative duration, less intraoperative bleeding, and shorter hospitalization [18]. Other uterine sparing minimally invasive techniques available for treating submucosal fibroids include uterine artery embolization (UAE) [19], hysteroscopic myomectomy after UAE [20], and transvaginal radio-frequency myolysis [21, 22] and magnetic resonance-guided high intensity focused ultrasound (MR-HIFU) [23, 24]. UAE showed improvement in symptom score and quality of life score outcomes when associated with heavy bleeding and submucosal leiomyoma location [25]. However, fertility and pregnancy concerns account significantly for the choice of therapy in women who desire uterine preservation. Fertility concerns explain the relative contraindications of ablation and embolization techniques in reproductive age women due to the risk of post-procedural ovarian failure [26] and inferior reproductive outcomes after these procedures compared to myomectomy [27].

The hysteroscopic myomectomy technique performed is with a mechanical hysteroscopic tissue removal system driven by their distinct advantages over the utilization of electrical current or laser energy with hysteroscopic removal of submucosal fibroids [28, 29]. The hysteroscopic myomectomy technique performed is with a mechanical hysteroscopic tissue removal system given their advantages. Either a reciprocating or rotating blade with suction is employed. The device incises the pathology by aspirating the submucosal leiomyoma and collects the tissue in a specimen bag within a vacuum container or fluid management system.

An additional benefit is that device insertion is only done once on initial entry through the operative port, decreasing the risk for perforation and increasing procedural efficiency. Moreover, mechanical hysteroscopic tissue removal system allows use of isotonic or electrolyte solutions such as sterile normal saline or lactated ringer, mitigating the risks associated with hypotonic solutions [30].

Although evaluation of the submucosal leiomyomas in terms of size, number, and location was tracked perioperatively, complete resection of submucosal leiomyoma(s) was not calculated. There was also no continuous monitoring of the size and number of sumucosal leiomyomas in the two study arms. Leiomyomas were classified according to the intramural extension of the submucosal leiomyoma using the European Society for Gynecological Endoscopy (ESGE) classification. A study limitation is the lack of correlation of the preoperative ESGE classification of intramural involvement with the QOL scores. Predictors for successful hysteroscopic myomectomy include small leiomyoma size, fewer intracavitary leiomyomas, and mainly intracavitary location [31, 32]. These factors deliver greater fibroid-related symptom resolution that could provide higher health-related quality of life scores. Since the study did not stratify analysis according to the type of submucosal leiomyoma, this precluded analysis of submucosal leiomyoma type compared to surgical success and outcome.

Although a study limitation is the small sample size, homogeneity of surgical technique and device use, which served to provide consistency in methodology and procedural skill as all cases were performed by a single surgeon, is a study strength. Another study strength is the use of a validated assessment tool, the patient-reported fibroid-related symptom severity and health-related quality-of-life (HR-QOL) questionnaire, with each patient acting as her own control.

Comparative effectiveness research (CER) is a directive of the Patient Protection and Affordable Care Act that was passed in 2010 to compare health outcomes and clinical effectiveness, risks, and benefits of two or more medical treatments, services, or clinical management and health practices [33]. Although the UFS-QOL questionnaire is a valid and reliable measure of patient-reported outcomes of fibroid related symptoms, these measures may not completely assess important qualities of a condition. This limitation may not clearly identify significant change in a particular disorder.

## Conclusion

Consideration of performing a multi-institutional study with a greater sample size could provide a better evaluation of treatment outcomes and increase generalizability of the results. While the medical treatment approach might be considered cost-effective as first-line therapy, hysteroscopic myomectomy offers long-term and sustained bleeding reduction in women with submucosal leiomyoma(s). Although there was no statistical difference in the change degree of improvement of overall quality of life among patients with symptomatic submucosal leiomyomas who received medical or surgical treatments in the study, there were greater differences in improvements in health-related quality of life scores over time after surgical treatment. Patients need to be counseled that either form of treatment is acceptable and result in a quality-of-life improvement. This could help guide patients who are considering hysteroscopic intervention for the treatment of submucosal leiomyomas.

## Data Availability

All data generated or analyzed during this study are included in this published article.

## Declarations

### What does this study add to the clinical work

? Patients with symptomatic submucosal leiomyomas who receive either medical or surgical treatment will have similar improvement of symptoms. But more considerable improvements occur in overall health-related quality of life scores after surgical therapy.
